# *Phalaenopsis LEAFY COTYLEDON1*-Induced Somatic Embryonic Structures Are Morphologically Distinct From Protocorm-Like Bodies

**DOI:** 10.3389/fpls.2019.01594

**Published:** 2019-11-29

**Authors:** Jhun-Chen Chen, Chii-Gong Tong, Hsiang-Yin Lin, Su-Chiung Fang

**Affiliations:** ^1^ Biotechnology Center in Southern Taiwan, Academia Sinica, Tainan, Taiwan; ^2^Agricultural Biotechnology Research Center, Academia Sinica, Taipei, Taiwan

**Keywords:** somatic embryogenesis, *Phalaenopsis* orchids, *LEAFY-COTYLEDON1*, protocorm-like-body, *Phalaenopsis equestris*

## Abstract

Somatic embryogenesis is commonly used for clonal propagation of a wide variety of plant species. Induction of protocorm-like-bodies (PLBs), which are capable of developing into individual plants, is a routine tissue culture-based practice for micropropagation of orchid plants. Even though PLBs are often regarded as somatic embryos, our recent study provides molecular evidence to argue that PLBs are not derived from somatic embryogenesis. Here, we report and characterize the somatic embryonic tissues induced by *Phalaenopsis aphrodite LEAFY COTYLEDON1* (*PaLEC1*) in *Phalaenopsis equestris*. We found that PaLEC1-induced somatic tissues are morphologically different from PLBs, supporting our molecular study that PLBs are not of somatic embryonic origin. The embryonic identity of PaLEC1-induced embryonic tissues was confirmed by expression of the embryonic-specific transcription factors *FUSCA3* (*FUS3*) and *ABSCISIC ACID INSENSITIVE3* (*ABI3*), and seed storage proteins *7S GLOBULIN* and *OLEOSIN*. Moreover, PaLEC1-GFP protein was found to be associated with the *Pa7S-1* and *PaFUS3* promoters containing the CCAAT element, supporting that PaLEC1 directly regulates embryo-specific processes to activate the somatic embryonic program in *P. equestris*. Despite diverse embryonic structures, PaLEC1-GFP-induced embryonic structures are pluripotent and capable of generating new shoots. Our study resolves the long-term debate on the developmental identity of PLB and suggests that somatic embryogenesis may be a useful approach to clonally propagate orchid seedlings.

## Introduction

Plant totipotency provides a means of clonally propagating a wide variety of plant species (Ikeuchi et al., 2013; Kareem et al., 2016; Radhakrishnan et al., 2017). *In vitro* regeneration bypasses the embryogenic program and directly generates seedlings with identical genetic makeup. One of the most common *in vitro* regeneration methods is somatic embryogenesis (Zimmerman, 1993; Pulianmackal et al., 2014). Somatic embryogenesis is crucial for establishing genetic transformation platforms for many non-model plant species and for clonal propagation of numerous high-value plants. For example, somatic embryos are used as transformation materials for alfalfa, American chestnut, cassava, cotton, grapevine, maize, mango, melon, Norway spruce, papaya, rose, tea tree, and walnut (Umbeck et al., 1987; Mcgranahan et al., 1988; Robertson et al., 1992; Fitch et al., 1993; Li et al., 1996; Brettschneider et al., 1997; Trinh et al., 1998; Mondal et al., 2001; Akasaka-Kennedy et al., 2004; Chavarri et al., 2004; Li et al., 2006; Polin et al., 2006; Vergne et al., 2010). In addition, the regeneration capacity of somatic embryos has made somatic embryogenesis a common method through which to clonally propagate economically important trees or herbal plants (Joshee et al., 2007; Nordine et al., 2014; Guan et al., 2016; Kim et al., 2019).

Embryogenesis is a defined developmental program during which the zygote grows and develops into a mature embryo. Somatic embryogenesis, on the other hand, activates the embryogenesis program in the absence of gamete fusion (von Arnold et al., 2002; Braybrook and Harada, 2008; Yang and Zhang, 2010; Feher, 2015). Zygotic embryogenesis and somatic embryogenesis programs not only share similar morphogenesis and maturation phases, they also share similar if not completely identical genetic and molecular networks (Zimmerman, 1993; Mordhorst et al., 2002; Gaj et al., 2005). Moreover, ectopic expression of several key embryo-associated transcription factors (TFs) is capable of inducing the embryogenesis program in somatic tissues (Lotan et al., 1998; Hecht et al., 2001; Stone et al., 2001; Boutilier et al., 2002; Zuo et al., 2002; Harding et al., 2003; Kwong et al., 2003; Gaj et al., 2005; Wang et al., 2009), demonstrating the developmental plasticity of plant tissues.

Orchids evolve specialized developmental programs including the co-evolution of diverse floral structures and pollinators (Waterman and Bidartondo, 2008), formation of pollen dispersal units (pollinia) (Pacini and Hesse, 2002), lack of cotyledon organogenesis during embryogenesis (Kull and Arditti, 2002; Yeung, 2017), and mycorrhizal fungi-assisted seed germination (Rasmussen, 2002), and all of these developmental processes contribute to their distinct morphology and physiological characteristics. These unique developmental strategies have not only fascinated many evolutionary and plant biologists; the beauty of the resulting floral structures is also enthusiastically admired by the general public. Much effort has been put into tissue culture-based clonal propagation of elite orchids over the past decades and this technology has transformed the orchid business into a multimillion-dollar orchid biotechnology industry (Winkelmann et al., 2006; Liao et al., 2011; Hossain et al., 2013).

Generally, embryogenesis of angiosperm plants starts from morphogenesis with continuous changes in embryo morphology and establishment of shoot-root polarity followed by maturation and desiccation processes (Bentsink and Koornneef, 2008; Braybrook and Harada, 2008). One of the characteristic features that defines the somatic embryo is the formation of the embryonic cotyledons. Even though orchid embryos go through a maturation and desiccation process, they lack characteristic cotyledons (organogenesis) and fail to establish a shoot-root axis during embryogenesis (Arditti, 1992; Dressler, 1993; Burger, 1998). Instead, a tubular embryo structure with an anterior meristem is formed. Upon germination, a tubular embryo emerges as a protocorm and new leaves and roots are generated from the anterior meristem of the protocorm (Nishimura, 1981).

Protocorm-like body (PLB)-based regeneration is commonly used to produce huge amounts of orchid seedlings of elite cultivars (Arditti and Krikorian, 1996; Chen et al., 2002; Arditti, 2009; Chugh et al., 2009; Yam and Arditti, 2009; Paek et al., 2011; Yam and Arditti, 2017). For years, much effort has been devoted to develop *in vitro* protocols to induce PLB and somatic embryo development either directly or indirectly (*via* the callus tissue) from explants to improve micropropagation in *Phalaenopsis* orchids (Tokuhara and Mii, 2001; Tokuhara and Mii, 2003; Kuo et al., 2005; Chen and Chang, 2006; Gow et al., 2009; Gow et al., 2010; Pramanik et al., 2016). PLBs are often induced from somatic tissues such as protocorms, floral stalk internodes, leaves, and root tips (Chen et al., 2002; Park et al., 2002; Chen and Chang, 2004; Chen and Chang, 2006; Teixeira da Silva et al., 2006; Zhao et al., 2008; Guo et al., 2010; Paek et al., 2011). Because embryogenesis produces tubular embryos, which, upon germination, develop into protocorms, and PLBs resemble protocorms morphologically, initiation, and development of PLBs is often regarded as somatic embryogenesis (Jones and Tisserat, 1990; Begum et al., 1994; Ishii et al., 1998). However, our recent study provides molecular evidence to argue that PLBs are not somatic embryos. Instead, PLB development seems to adopt a shoot pole-related organogenesis program (Fang et al., 2016). So if PLBs are not somatic embryos, what does the somatic embryo of orchids look like? Are somatic embryos of orchid species also prolific in propagating clonal plants? To address these questions and to explore the potential of using orchid somatic embryos for clonal propagation, we generated transgenic plants overexpressing *Phalaenopsis aphrodite LEAFY COTYLEDON1* (*PaLEC1*) gene in *Phalaenopsis equestris*. *LEC1* encodes a HAP3 subunit of CCAAT-binding TF required for embryo development (Lotan et al., 1998). Moreover, ectopic expression of *LEC1* isolated from *Arabidopsis* and other plant species has been demonstrated to be a potent inducer of the somatic embryogenesis program (Lotan et al., 1998; Yazawa et al., 2004; Garcês et al., 2014; Zhu et al., 2014; Min et al., 2015; Fang et al., 2016). Consistently, we have shown that *PaLEC1* is capable of inducing somatic embryogenesis in *Arabidopsis* (Fang et al., 2016). Here, we report *PaLEC1*-induced embryonic tissues in *P. equestris* and demonstrate their embryonic identity by validation of expression of the embryonic-specific genes. To the best of our knowledge, this is the first time that somatic embryonic tissues was shown to be induced by the embryonic factor, *PaLEC1*, in the transgenic orchid plants. Our studies reveal that *PaLEC1* may be a useful tool for large scale propagation of clonal plants in orchid biotechnology.

## Materials and Methods

### Plant Materials and Growth Conditions

*P. equestris* plants were grown and maintained in a growth chamber with alternating 12 h light (23°C)/12 h dark (18°C) cycles. Hand pollination was conducted for each flower, as described previously (Chen and Fang, 2016).

Seeds were allowed to germinate in germination medium (0.1% tryptone, 1.8% sucrose, 2% potato, 0.75% agar, 1.08 g/l MS salt, 1X MS vitamin pH 5.7). For regeneration assay, the protocorm segments (explants) were transferred to T2 medium containing 0.1% (w/v) tryptone, 2% (w/v) sucrose, 2% (w/v) potato homogenate, 2.5% (w/v) banana homogenate, 0.01% (v/v) citric acid, 0.1% (w/v) charcoal, and 1% (w/v) agar adjusted to pH 5.5 (Chen et al., 2009) to induce PLBs.

### Generation of Transgenic *Phalaenopsis equestris* Plants

Protocorms or PLBs of *P. equestris* were used for transformation. *Agrobacterium*-based transformation was conducted as described previously with slight modification (Liau et al., 2003). Briefly, protocorms or PLBs were co-cultured with *Agrobacterium tumefaciens* strain EHA105 culture (OD_600_ = ~ 0.8) containing the *35S:PaLEC1-GFP* or pH7FWG2 (vector only control) construct in the presence of 100 μM acetosyringone overnight (for protocorms) or 3 days (for PLBs) in the dark. After removing *Agrobacterium* inoculum, the infected tissues were washed four to six times with distilled water containing 40 mg/L meropenem (China Chemical & Pharmaceutical). The infected tissues were allowed to recover in New Dogashima medium (NDM) (Belarmino and Mii, 2000) containing 10 g/L maltose, 0.1 mg/L naphthaleneacetic acid (NAA), and 0.4 mg/L benzyladenine (BA) and supplemented with 40 mg/L meropenem for 1 week in the dark. All the NDM media used in this study contained 10 g/L maltose, 0.1 mg/L NAA, and 0.4 mg/L BA. The recovered tissues were then selected on NDM agar plates supplemented with 20 mg/L hygromycin and 40 mg/L meropenem at 23°C in the dark. The infected tissues were selected continuously by sub-culturing to fresh NDM plates supplemented with 20 mg/L hygromycin and 40 mg/L meropenem at 2- to 4-week intervals until new leaves emerged. The hygromycin-resistant transgenic plants were then transferred to T2 (Chen et al., 2009) medium supplemented with 20 mg/L hygromycin and 40 mg/L meropenem to encourage growth.

### Deoxyribonucleic Acid Isolation and Southern Blotting

Genomic DNA from young seedlings (1 to 1.5 cm in size) of T1 transgenic plants and wild-type plants was isolated by cetyltrimethylammonium bromide (CTAB)-based method (Murray and Thompson, 1980; Saghai-Maroof et al., 1984) with some modifications. Briefly, freeze-dried tissue was ground in the presence of liquid nitrogen, resuspended in 1 g/10 ml of the CTAB extraction buffer [100 mM Tris, pH 8.0, 1.4 M NaCl, 20 mM EDTA, 2% CTAB, 0.2% (w/v) polyvinylpyrrolidone 40, and 0.2% β-mercaptoethanol], and incubated at 65^o^C for 60 min with occasional gentle vortexing. Equal volume of chloroform:isoamylalcohol (24:1) was then added and mixed by inversion for 15 min. Plant extract was then centrifuged at 3220 × g for 15 min at 4^o^C. The aqueous phase was extracted with equal volume of chloroform:isoamylalcohol (24:1) one more time. The aqueous phase was transferred to a new tube and equal volume of isopropanol was added, mixed, and incubated at ‑20^o^C overnight. DNA was precipitated by centrifugation at 3,220 × g at 4^o^C for 10 min. DNA was washed once with 75% ethanol followed by 99% ethanol. DNA was then resuspended in H_2_O supplemented with 10 µg/ml RNase A and incubated at 37^o^C overnight to remove RNA. This method yielded approximately 150‑350 µg per g of orchid tissue. Approximately 15 µg DNA was digested and Southern blotting and hybridization were carried out as described previously (Fang et al., 2006).

### Antibody Generation and Western Blotting

To generate polyclonal antibodies specific for PaLEC1 protein, PaLEC1 peptide YLHRYRELEGDHRGSIRG (LTK BioLaboratories, Taiwan) was synthesized to generate rabbit polyclonal antisera. Polyclonal antibodies raised against PaLEC1 were affinity purified for western blotting analysis.

To prepare total protein extract, tissues were ground to a fine powder in the presence of liquid nitrogen using a pestle and mortar. One gram of ground tissues was homogenized in 1 ml 1X Urea-based protein extraction buffer (250 mM Tris-HCl, pH 6.8, 3.5% sodium dodecyl sulfate (SDS), 10% glycerol, 1 M urea) supplemented with 1X protease inhibitor cocktail (Sigma-Aldrich), 1 mM *phenylmethylsulfonyl fluoride* by vortexing. Cell debris was removed by centrifugation at 16,100 × g for 10 min at 4°C. Concentration of protein lysates was determined by Bio-Rad DC Protein Assay Kit (Bio-Rad).

Protein lysates were resolved by a standard 10% SDS-polyacrylamide gel electrophoresis (PAGE) and transferred to Immobilon-P PVDF membrane (Merck Millipore). Blots were blocked in 1 × TBST (20 mM Tris base, 150 mM NaCl, 0.1% Tween 20) supplemented with 5% non-fat milk at room temperature (RT) for 1 h and incubated with anti-GFP antibodies (Roche) diluted 1:2,000 in 1 × TBST supplemented with 5% non-fat milk at 4°C overnight. For detection of PaLEC1 and PaRbCL proteins, blots were incubated with PaLEC1 (1:2,000) and RbCL (1:5,000, Agrisera) antibodies diluted in 1 × TBST with 5% non-fat milk for 1 h at RT. Blots were washed three times for 15 min each time and then incubated with horseradish peroxidase (HRP) conjugated goat-anti-mouse-IgG (1:20,000, Jackson ImmunoResearch Laboratories), or goat-anti-rabbit-IgG (1:20,000, PerkinElmer) for 1 h at RT. After washing in 1 × TBST for 15 min three times, the blots were processed for chemiluminescence detection using Amersham ECL select Western Blotting detection reagent. The images were acquired by a ChemiDoc XRS+ imager (Bio-Rad).

### Ribonucleic Acid Isolation and Quantitative Real-Time Polymerase Chain Reaction

Plant tissues (young seedlings or callus tissues) were homogenized by pestle and mortar in the presence of liquid nitrogen. RNA was isolated using RNA extraction reagent (3-Zol, MDBio) according to the manufacturer’s instructions. Total RNA was treated with RNase-free DNase (Qiagen) to remove DNA followed by RNeasy column purification (Qiagen) according to the manufacturer’s instructions.

Approximately 5 μg DNA-free RNA was reverse transcribed in the presence of a mixture of oligo dT and random primers (9:1 ratio) using the GoScript Reverse Transcription System (Promega) according to the manufacturer’s instructions. Ten microliters of quantitative RT-PCR reaction was set up as follows: 2.5 μl of 1/20 diluted complementary DNA, 0.2 μM of primers, and 5 μl of 2X KAPA SYBR FAST master mix (KAPA Biosystems). The PCR program was used for DNA amplification: 95°C for 1 min, 40 cycles of 95°C for 5 s, and 58°C, 60°C, or 62°C for 20 s. PCR was performed in triplicate. Standard error was calculated from three technical replicates. Fold change in expression was calculated as 2 ^‑△△CT^. A melting curve of each PCR was examined to ensure no spurious products were present. Primer pairs used for quantitative PCR are listed in **Supplementary Table S1**. A ubiquitin gene, *PaUBI* (Lin et al., 2014), was used as an internal control.

### Light Microscopy

Tissues were fixed in paraformaldehyde fixative and sectioned to 10 μm in thickness using the hybrid-cut method as previously described (Chen et al., 2016). Tissues sections were then stained with hematoxylin solution and photographed on a Zeiss Axio Scope A1 microscope equipped with an Axio-Cam HRc camera (Zeiss, Germany).

Fresh tissues of transgenic plants were hand sectioned and fluorescence images were photographed on a LSM 710 Confocal Microscope (Zeiss).

### Chromatin Immunoprecipitation-Polymerase Chain Reaction

Chromatin immunoprecipitation was conducted as described previously (Villar and Köhler, 2010) with slight modification. One gram of young seedling (1 to 1.5 cm in size, pooled from approximately 10 T1 seedlings) tissue was fixed in 37 ml of 1% formaldehyde and vacuum-infiltrated for 20 min. Two and half milliliters of 2 M glycine was added to stop crosslinking. Fixed tissues were then homogenized by pestle and mortar and chromatin was extracted and filtered by a 70 μM cell strainer (Biologix Group Limited) as described (Villar and Köhler, 2010). Chromatin was resuspended in 200 μl of nuclei lysis buffer (50 mM Tris-HCl, pH 8.0; 10 mM EDTA, 1% SDS, and 1X protease inhibitor cocktail) before sonication. Chromatin was then transferred to a micro TUBE-500 AFA Fiber Screw-Cap tube and sonicated for 12 min or 20 min under the condition: peak incident power 150 S; duty factor 2%, cycles per burst 200, in a Covaris S220 system (Covaris). The sheered chromatin was precleared by incubating with 40 μl protein A Magnetic Sepharose beads (Merck) at 4°C for 1.5 h. In the meantime, 5 μg PaLEC1 polyclonal antibody or histone H3 antibody (Agrisera) was incubated with protein A beads at 4°C for 1.5 h. After preclearing, protein A/antibody mix was added to each sheered chromatin sample and incubated at 4°C overnight. Magnetic beads were then washed as described (Villar and Köhler, 2010) and eluted by adding 150 μl of elution buffer (1% SDS and 0.1% NaHCO_3_) and incubating at 65°C for 15 min. Elution was repeated one more time and elutes were pooled into a single tube. Twelve microliters of 5 M NaCl was added into 200 μl elutes and incubated at 65°C overnight. Proteins were removed by incubating with 6 μl of 2 mg/ml proteinase K, 6 μl of 0.5 M EDTA, and 12 μl of 1 M This-HCl (pH 6.5) at 45°C for 2 h. Immunoprecipated DNA was then purified by QIAquick PCR Purification Kit (Qiagen) following the manufacturer’s instructions.

Twenty microliters of PCR reaction was set up as follows: 2 or 3 μl of chromatin immunoprecipitation (ChIP) DNA, 0.5 μM of primers, 2 μl of 10X PCR buffer, 3% dimethyl sulfoxide, and 1.25 unit Power Taq polymerase (Genomics, Taiwan). The PCR program was used for amplification of immunoprecipitated DNA: 95°C for 2 min, 42 cycles of 94°C for 5 s, 58–60°C for 20 s, and 72°C for 20 s. Primers used for ChIP-PCR are listed in **Supplementary Table S1**. ChIP-PCR was repeated three times using three independent biological samples.

## Results

### Generation of Transgenic *Phalaenopsis equestris* Plants Overexpressing *PaLEC1-GFP*

To explore the possibility of inducing a somatic embryonic program in *Phalaenopsis* orchids and to investigate whether somatic embryonic potential can be utilized for micropropagation, transgenic *P. equestris* plants overexpressing the embryonic maker *PaLEC1* that has been shown to induce somatic embryogenesis in *Arabidopsis* (Fang et al., 2016) were generated. After transformation, we successfully obtained multiple independent transgenic lines carrying the *35S:PaLEC1-GFP* transgene with the hygromycin phosphotransferase (hpt) marker (**Supplementary Figure S1A**). Expression of PaLEC1-GFP was also confirmed in four out of seven independent transgenic lines by western blotting using the GFP antibody (**Supplementary Figure S1B**). However, multiple transformation attempts failed to generate transgenic plants carrying the empty pH7FWG2 vector. It is not clear why only *35S:PaLEC1-GFP* transgenic plants were obtained. Overexpression of the embryonic genes *BABYBOOM* has been reported to enhance embryogenic callus proliferation, overall transformation efficiency, and regeneration capacity in several monocot plants recalcitrant for transformation (Lowe et al., 2016). Hence, it is possible that ectopic PaLEC1 enhances transformation efficiency by increasing embryogenic callus formation and regeneration ability.

### Altered Protocorm Development in Transgenic Plants Overexpressing *PaLEC1-GFP*

The *35S:PaLEC1-GFP* transgenic plants were allowed to grow in growth chamber for approximately 5 years before reaching maturity for flowering. *35S:PaLEC1-GFP* transgenic lines #1, #2, and #3 started to flower in March of 2018 and were therefore used in this study. The flowers were hand-pollinated and T1 seeds were allowed to produce in developing capsules. Seeds were sown and allowed to germinate on medium containing hygromycin.

**Figure 1:**
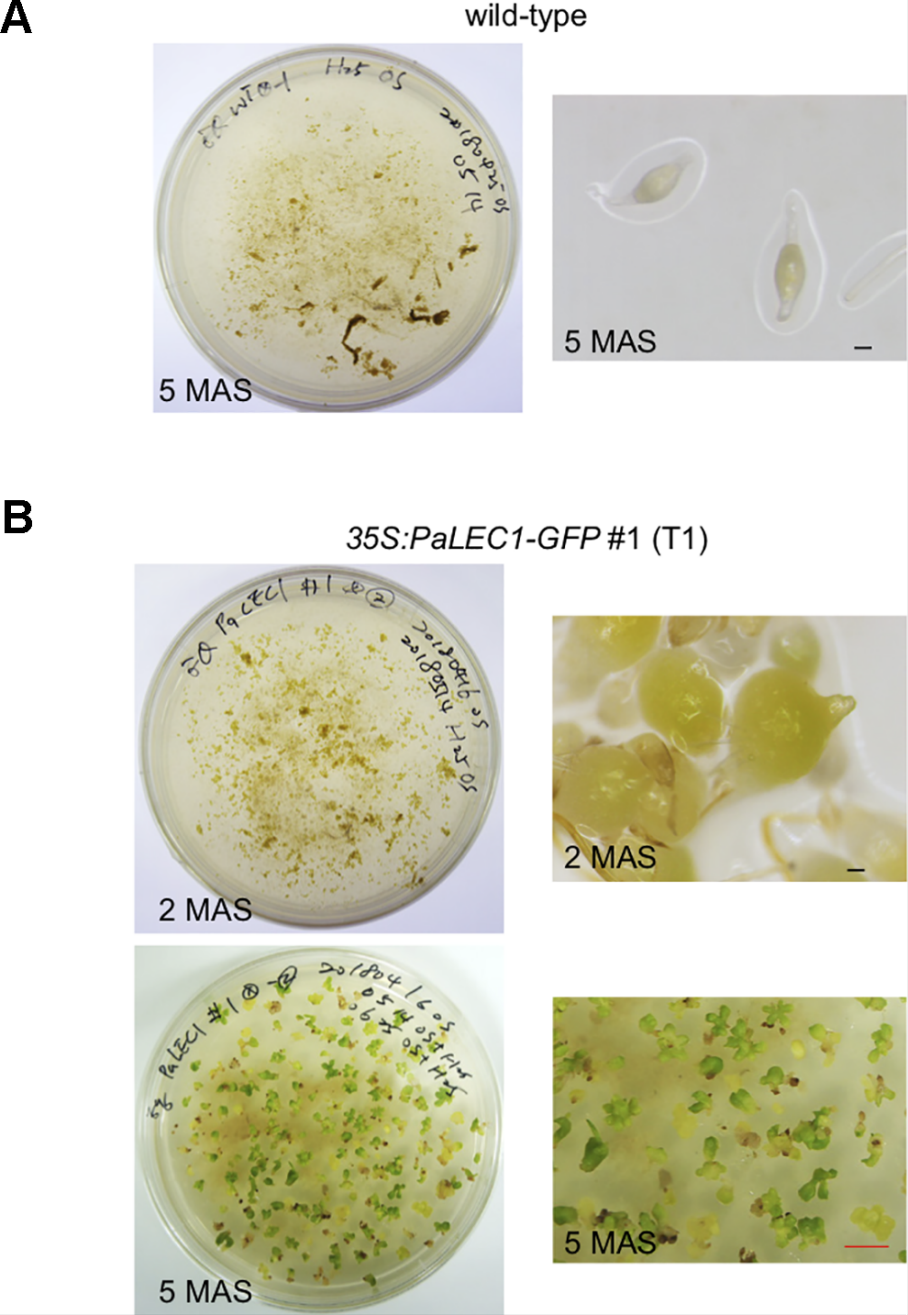
Selection of T1 *35:PaLEC1-GFP #1* transgenic plants on hygromycin plates. **(A)** Wild-type protocorms failed to develop on hygromycin-containing medium. **(B)** *35:PaLEC1-GFP #1* protocorms continued to grow and developed into seedlings 5 months after sowing. MAS, month after sowing. Black scale bar = 100 μm. Red scale bar = 0.5 cm.

As expected, seeds harvested from wild-type control plants failed to grow and were arrested on hygromycin-containing plates 5 months after sowing (MAS, **Figure 1A**). T1 seeds from *35S:PaLEC1-GFP* transgenic lines #1 and #2, on the other hand, started to expand and turned green at 2 MAS (**Figure 1B**). The germinated protocorms continued to grow and produce new shoots on hygromycin-containing plates at 5 MAS (**Figure 1B**). Transgenic *35S:PaLEC1-GFP* line #3 failed to produce viable seeds and was therefore omitted from further study. Southern blotting was used to confirm independent integration of the transgene in *35S:PaLEC1-GFP* #1 and *35S:PaLEC1-GFP* #2 lines (**Supplementary Figure S1C**).

**Figure 2:**
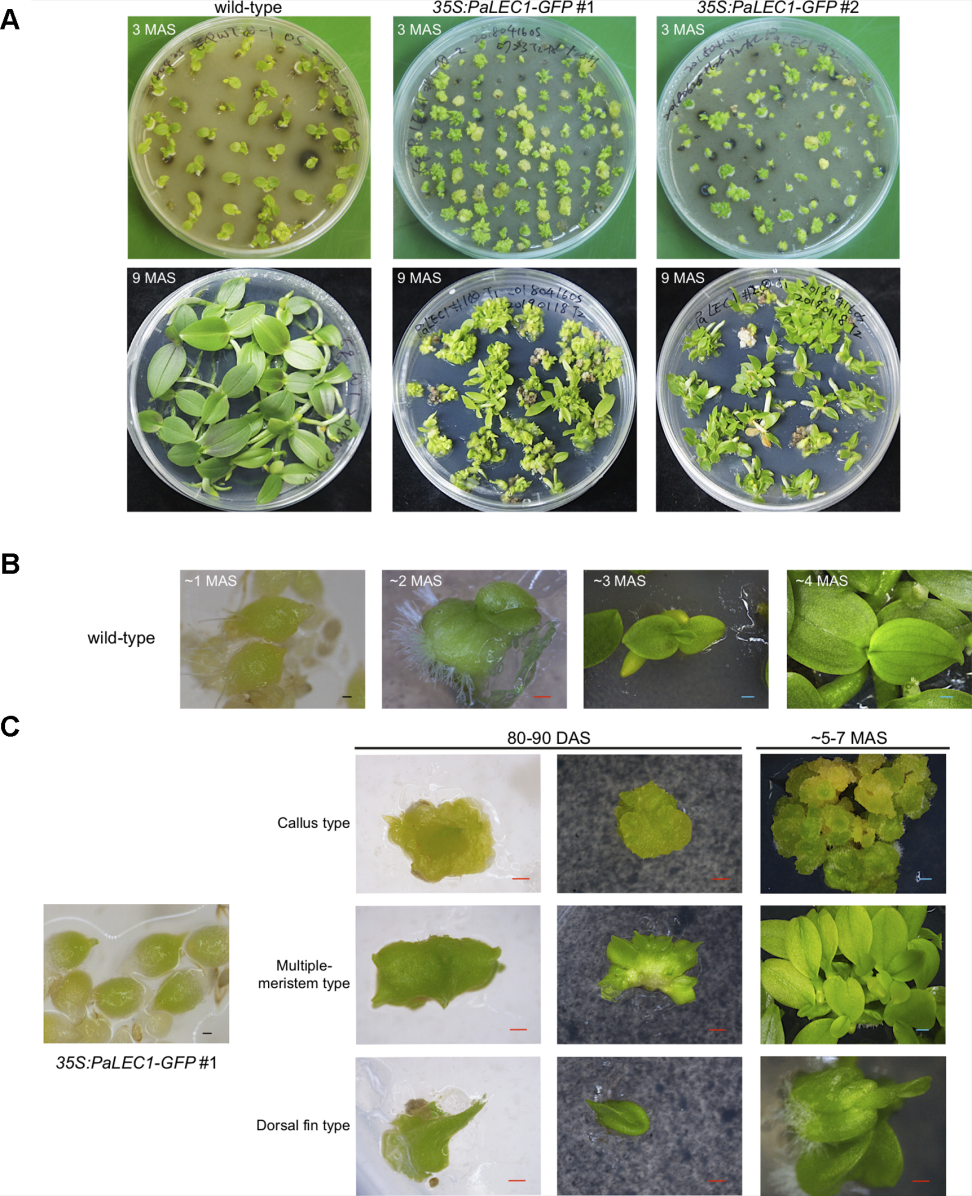
Developmental processes of T1 *35:PaLEC1-GFP #1* transgenic plants. **(A)** Developing seedlings of wild-type plant, *35:PaLEC1-GFP #1*, and *35:PaLEC1-GFP #2* transgenic plants at 3 and 9 months after germination. **(B)** Germinated protocorms of wild-type plants developed into young seedlings at 1, 2, 3, and 4 months after germination. **(C)** Germinated protocorms of *35:PaLEC1-GFP #1* transgenic plants developed into callus-type, multiple-meristem type, and dorsal fin-like structures. MAS, month after sowing; DAS, day after sowing; Red scale bar = 500 μm. Blue scale bar = 1 mm.

Protocorms from wild-type plants continued to develop and grow into young seedlings at approximately 80–90 days after sowing (DAS, **Figures 2A**, **B**). Instead of growing into individual seedlings, protocorms of the transgenic *35S:PaLEC1-GFP* #1 plants developed into various structures (**Figure 2A**) that did not completely resemble seedlings of wild-type seedlings at 80–90 DAS (**Table 1**). For example, approximately 25% germinated protocorms underwent uncontrolled cell division and formed unorganized callus-like structures (callus type, **Figure 2C**), which is similar to somatic embryonic calli commonly observed in other plant species (Zimmerman, 1993; Yang and Zhang, 2010; Ikeuchi et al., 2013). Around 48% of protocorms, on the other hand, developed into a fan-like structure with multiple meristems forming on the surface (multiple-meristem type, **Figure 2C**). Around 27% protocorms formed a dorsal-fin-like structure with newly emerging leaves growing from it (dorsal-fin type, **Figure 2C**). The callus-like structure was capable of acquiring meristematic fate and developed into shoots (**Supplementary Figure S2**). Even though only one shoot was developed from the dorsal-fin type structure, new shoots could occasionally emerge from the base of the dorsal-fin type structure.

**Table 1:** Different types of structures were formed from germinating protocorms of *35S:PaLEC1-GFP* transgenic lines. Number of the represented type (number) and percentage of the represented type (%) of structures are shown.

	wt		*35S:PaLEC1-GFP#1*		*35S:PaLEC1-GFP#2*	
	Number	%	Number	%	Number	%
Wt seedling	32	94.1%	0	0	25	35.2%
Callus-like	0	0	27	25.0%	6	8.5%
Multiple meristem-like	0	0	52	48.1%	40	56.3%
Dorsal fin-like	0	0	29	26.9%	0	0
PLB	2	5.9%	0	0	0	0
**Total**	34	100%	108	100%	71	100%

**Figure 3:**
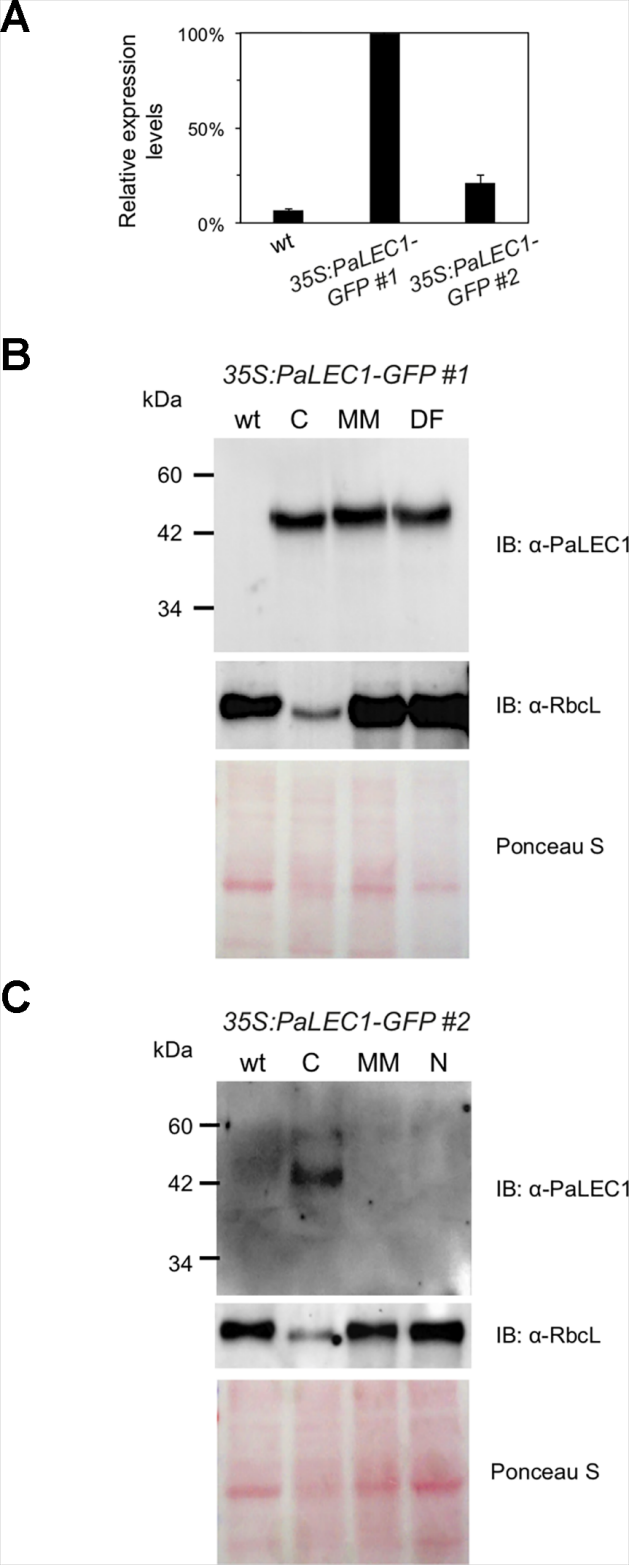
Expression of PaLEC1-GFP protein in *35:PaLEC1-GFP* transgenic lines. **(A)** Quantitative RT-PCR analysis of expression of *PaLEC1-GFP* in wild-type (wt) and two *35:PaLEC1-GFP* transgenic lines. **(B)** Western blotting showing expression of PaLEC1-GFP and PaRbcL proteins in callus-type, multiple-meristem type, and dorsal fin-like embryonic tissues of *35:PaLEC1-GFP*#1 plants. **(C)** Western blotting showing expression of PaLEC1-GFP and PaRbcL proteins in callus-type, embryonic tissues of *35:PaLEC1-GFP*#2 plants. Blots stained with Ponceau S staining were used to visualize total loaded protein. wt, wild-type plants; C, callus-like tissues; MM, multiple meristem-like structures; DF, dorsal fin-like structures; N, normal wt-like tissues.

Even though PaLEC1-GFP protein could not be detected in T0 *35S:PaLEC1-GFP* #2 transgenic plants, some of the germinated T1 seeds also developed into structures that were similar to those observed in *35S:PaLEC1-GFP* #1 line 80–90 DAS (**Supplementary Figure S3**). Approximately 35.2% of protocorms grew and became normal seedlings as seen for wild-type protocorms (**Table 1**). Around 56.3% protocorms developed into multiple-meristem type structures and 8.5% protocorms developed into callus-like structures. No dorsal-fin type structure was observed in the T1 *35S:PaLEC1-GFP* #1 line. Quantitative RT-PCR and western blotting were conducted to validate and re-evaluate the expression of *PaLEC1-GFP* mRNA and its encoded protein in *35S:PaLEC1-GFP* #1 and *35S:PaLEC1-GFP* #2. As compared to *35S:PaLEC1-GFP* #1 seedlings, *35S:PaLEC1-GFP* #2 had much less *PaLEC1-GFP* mRNA than *35S:PaLEC1-GFP* #1 line (**Figure 3**). Because GFP monoclonal antibody may be less sensitive than polyclonal antibodies in detecting PaLEC1-GFP protein, PaLEC1 polyclonal antibodies were generated. Similar to hygromycin-resistant T0 *35S:PaLEC1-GFP* #1 parent, PaLEC1-GFP protein was expressed in high abundance in callus-like, multiple-meristem, and dorsal-fin types of structures germinated from T1 seeds (**Figure 3**). Using the PaLEC1 polyclonal antibodies, PaLEC1-GFP protein was also detected in callus-like tissues but was barely detectable in multiple-meristem-like structures and wild-type-like seedlings of *35S:PaLEC1-GFP* #2 transgenic line (**Figure 3**), indicating PaLEC1-GFP protein was expressed but accumulated in very low abundance. We noticed that PaLEC1-induced callus tissues had a reduced amount of ribulose bisphosphate carboxylase large subunit (RbcL) protein (**Figure 3**), that is consistent with the pale-yellow appearance of PaLEC1-induced callus tissues.

**Figure 4:**
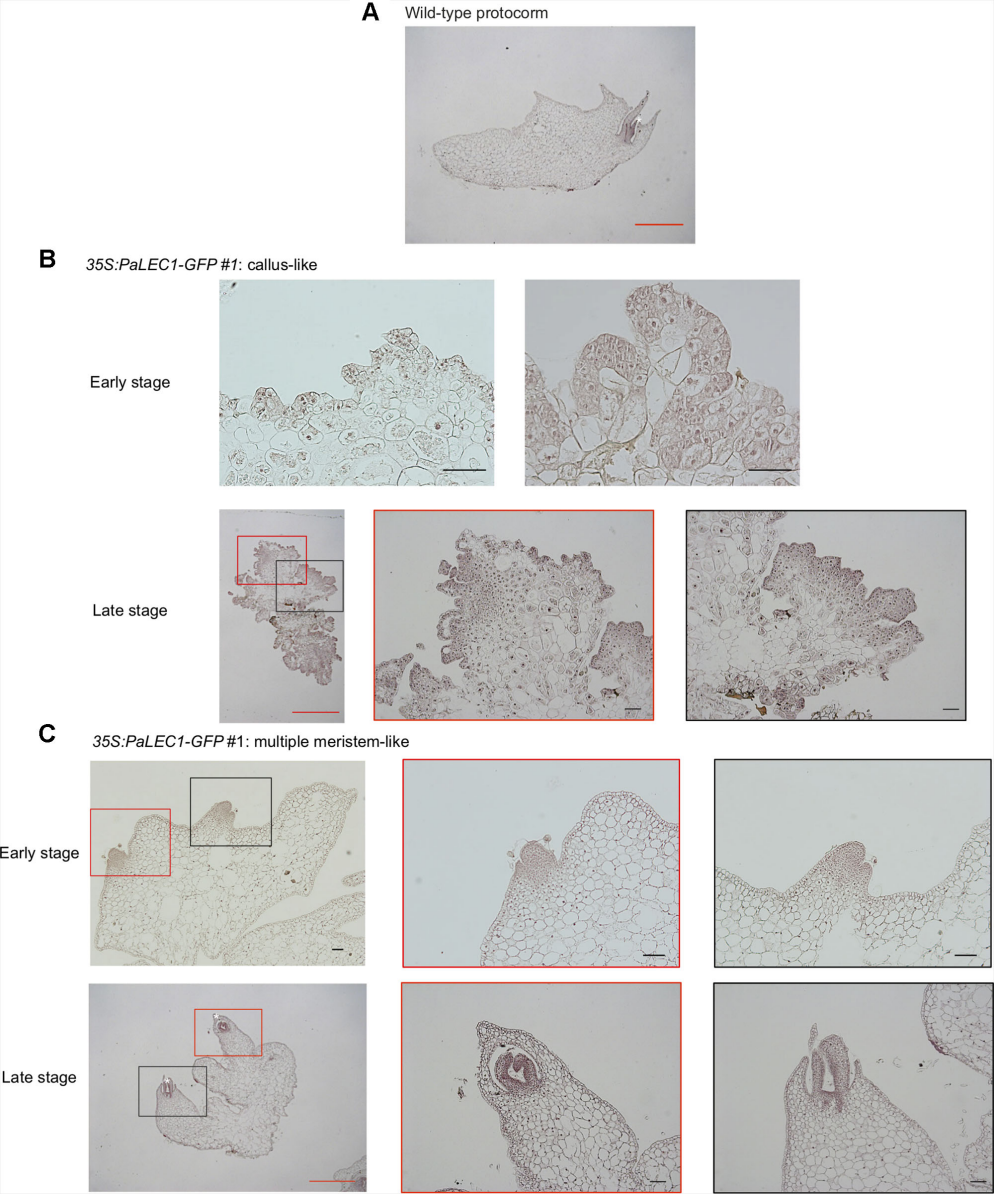
Histological examination of embryonic tissues of *35:PaLEC1-GFP#1* transgenic plants. **(A)** Light micrograph of 10 μm longitudinal section of developing wild-type protocorm stained with hematoxylin. **(B)** Light micrograph of a 10 μm section of the early and late stages of callus-like tissues derived from protocorms of the T1 *35:PaLEC1-GFP#1* seeds. **(C)** Light micrograph of a 10 μm section of the early and late stages of multiple meristem-like tissues derived from protocorms of the T1 *35:PaLEC1-GFP#1* seeds. Shoot apical meristem (SAM) with the emerging leaf primordia are marked by a white asterisk. For panel **(B** and **C)**, low and high magnification of structures marked with red and black rectangles are shown. Red scale bar = 1 mm. Black scale bar = 100 μm.

To gain further structural details of the PaLEC1-induced structures, histological section and histochemical staining were conducted. As described previously (Yeung, 2017), shoot apical meristem was initiated from the apical end of a protocorm in the wild-type plants (**Figure 4**). In the *35S:PaLEC1-GFP* transgenic plants, active cell division appeared to take place on the outer cell layer (epidermal cells) of developing protocorms (early stage, **Figure 4**). Protocorms then grew into friable and unstructured cellular masses with small cells showing dense cytoplasm on the surface (late stage, **Figure 4**). As for multiple meristem-type structures, the dividing cells on the surface of developing protocorms gradually organized into the shoot apical meristems at the early stage (**Figure 4**). As development proceeded, leaf primordia started to emerge from the organized shoot apical meristems (late stage, **Figure 4**) and multiple shoots were formed.

### Explants of Transgenic Plants Overexpressing *PaLEC1-GPF* Induce Tissues That Are Dissimilar to Protocorm-Like Bodies

**Figure 5:**
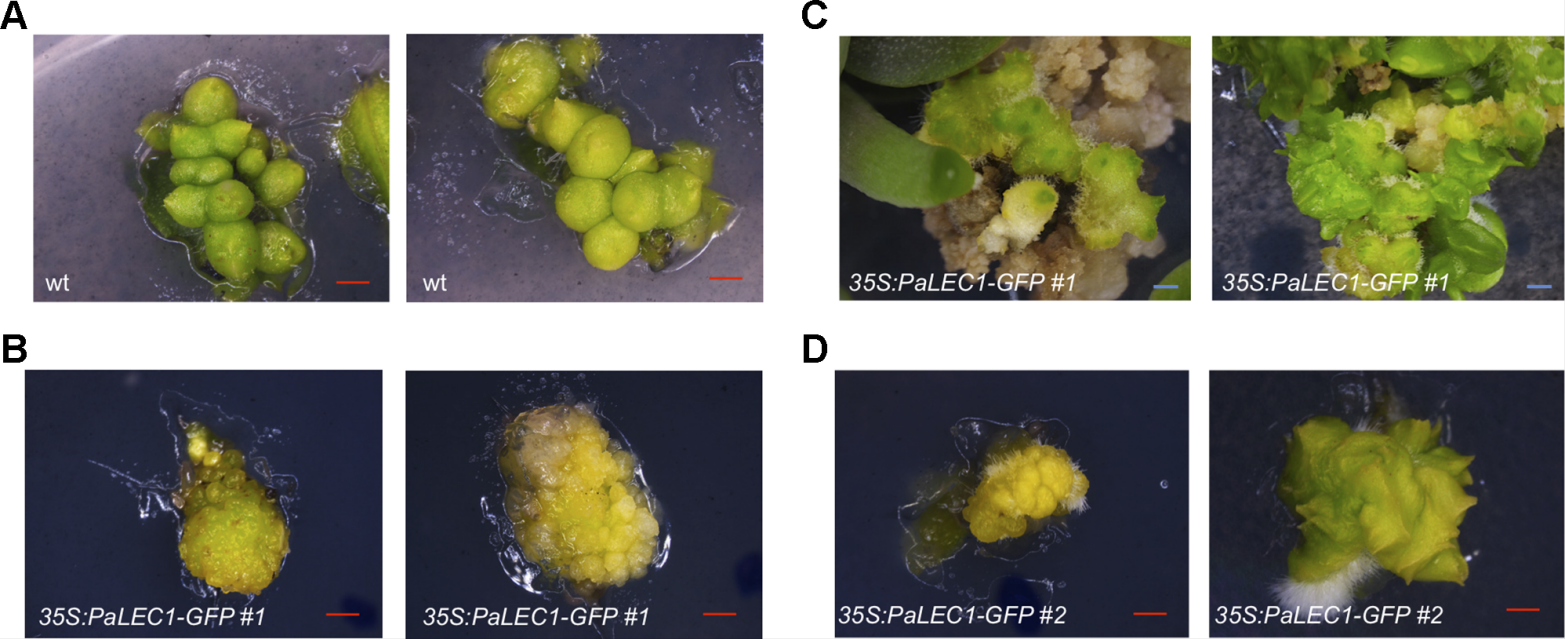
Clonal propagation from protocorm-based explants of *35:PaLEC1-GFP#1* transgenic plants. **(A)** PLBs generated from cutting of wild-type protocorms. **(B)** Callus-like tissues produced by cutting of *35:PaLEC1-GFP#1* protocorms. **(C)** Callus-like tissues generated from *35:PaLEC1-GFP#1* grew into multiple-shoot like structures. **(D)** Callus-like and multiple shoot-like tissues produced by cutting of *35:PaLEC1-GFP#2* protocorms. For each panel, two independent clusters of tissues were photographed and showed. Red scale bar = 500 μm. Blue scale bar = 1 mm.

Overexpression of *LEC1* gene is known to be a potent inducer of somatic embryos (Lotan et al., 1998; Mu et al., 2008; Horstman et al., 2017a). We were therefore interested in asking what types of somatic embryonic structures could be induced by *PaLEC1* in *P. equestris*. To do so, protocorms were used as the explants for induction of somatic embryonic tissues. As described previously (Zhao et al., 2008; Mahendran and Bai, 2012; Fang et al., 2016), cutting protocorms of wild-type plants induced green PLBs (**Figure 5**). Cutting protocorms of T1 *35S:PaLEC1-GFP* #1 transgenic line, on the other hand, induced callus-like tissues and pale yellow-green protrusions (**Figure 5**). The appearance of pale yellow-green color is consistent with a previous report stating that overexpressing *LEC1* downregulates light responsive genes in *Arabidopsis* (Junker et al., 2012). Protrusions and callus-like tissues continued to grow and develop into unorganized thick and flat tissues, which then eventually grew into multiple-meristem type structures that were capable of producing multiple shoots (**Figure 5**). Using protocorms of T1 *35S:PaLEC1-GFP* #2 line as explants also induced callus-like and multiple-meristem type structures (**Figure 5**). Taken together, we conclude that *PaLEC1*-induced tissues have the ability to establish new shoot apical meristems for shoot regeneration.

To evaluate whether PaLEC1-GFP protein was regulated post-transcriptionally as somatic embryonic calli programmed to initiate shoot formation, PaLEC1-GFP protein was monitored by western blotting. As shown in **Supplementary Figure S4**, PaLEC1-GFP protein remained stable throughout the process.

### PaLEC1 Is Sufficient to Activate Embryonic Marker Genes in Seedlings of *PaLEC1-GPF* Transgenic Plants

**Figure 6:**
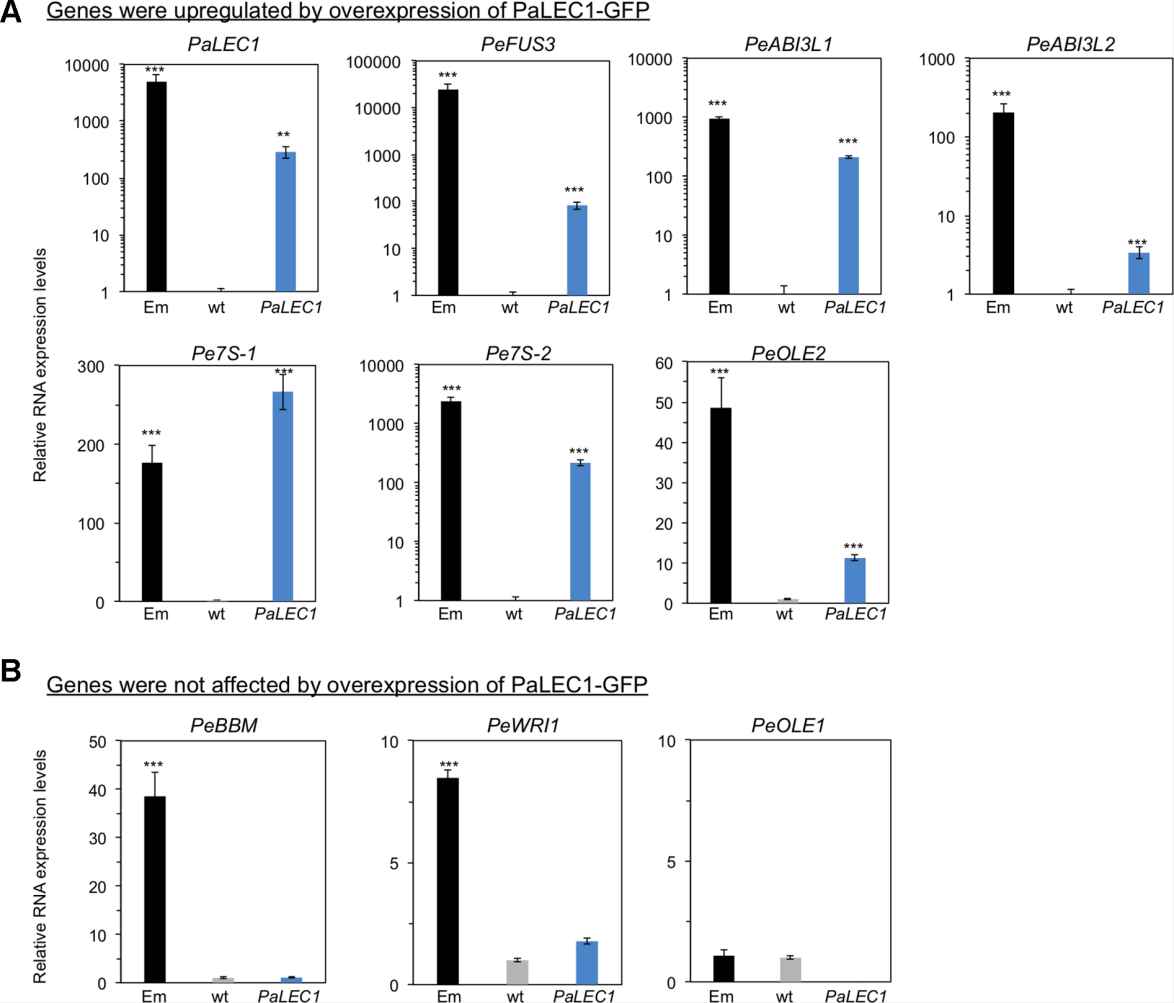
Expression of embryonic genes in *35:PaLEC1-GFP#1* transgenic plants. **(A)** Expression of indicated embryonic genes was induced in *35:PaLEC1-GFP#1* transgenic plants. **(B)** Expression of indicated embryonic genes was not induced significantly by overexpression of PaLEC1-GFP protein. Em, zygotic embryonic tissues collected at 130 day after pollination. wt, wild-type plant. *PaLEC1*, *35:PaLEC1-GFP#1* transgenic plants. ***, *P* < 0.001; **, *P* < 0.01. Three independent biological replicates were performed.

To evaluate the embryonic identity of PaLEC1-GFP overexpressing somatic tissues, expression of previously identified embryonic-specific genes (Fang et al., 2016) were examined in T1 *35S:PaLEC1-GFP* #1 protocorm-derived young seedling tissues. Expression of the embryonic TFs *ABSCISIC ACID INSENSITITVE3-like1* (*PeABI3L1*), *PeABI3L2, BABY BOOM* (*PeBBM*), *FUSCA3* (*PeFUS3*), and *WRINKLED1* (*PeWRI1*) and seed storage proteins, *7S GLOBULIN1* (*Pe7S-1*), *Pe7S-2*, *OLEOCIN1* (*PeOLE1*), and *PeOLE2* genes were confirmed to be preferentially expressed in ovary tissues collected at 130 days after pollination (DAP) of *P. equestris* (**Figure 6**). As expected, overexpression of PaLEC1-GFP caused significant induction of embryonic TFs *PeFUS3* and *PeABI3L1*, and seed storage proteins, *Pe7S-1*, *Pe7S-2*, and *PeOLE2* genes (**Figure 6**). Overexpression of PaLEC1-GFP protein only led to mild elevation of *PeABI3L2* mRNA and did not affect expression of *PeBBM*, *PeWRI1*, and *PeOLE1* genes (**Figure 6**), suggesting that PaLEC1 activates only part of the embryonic program. Similar expression patterns were also confirmed in independently collected young seedlings and callus tissues except *PeABI3L2* whose expression was not induced in PaLEC1-induced callus tissues (**Supplementary Figures S5A**, **B**). It is likely that the moderate level of *PaLEC1-GFP* (**Supplementary Figure S5B**) in callus was not enough to induce *PeABI3L2*. Activation of *PeFUS3* was also confirmed in leaf tissues of five independent T0 *35S:PaLEC1-GFP* transgenic plants (**Supplementary Figure S6**).

### PaLEC1 Is Predominantly Localized in the Nucleus and Is Associated With Target Promoters Containing the CCAAT Element

**Figure 7:**
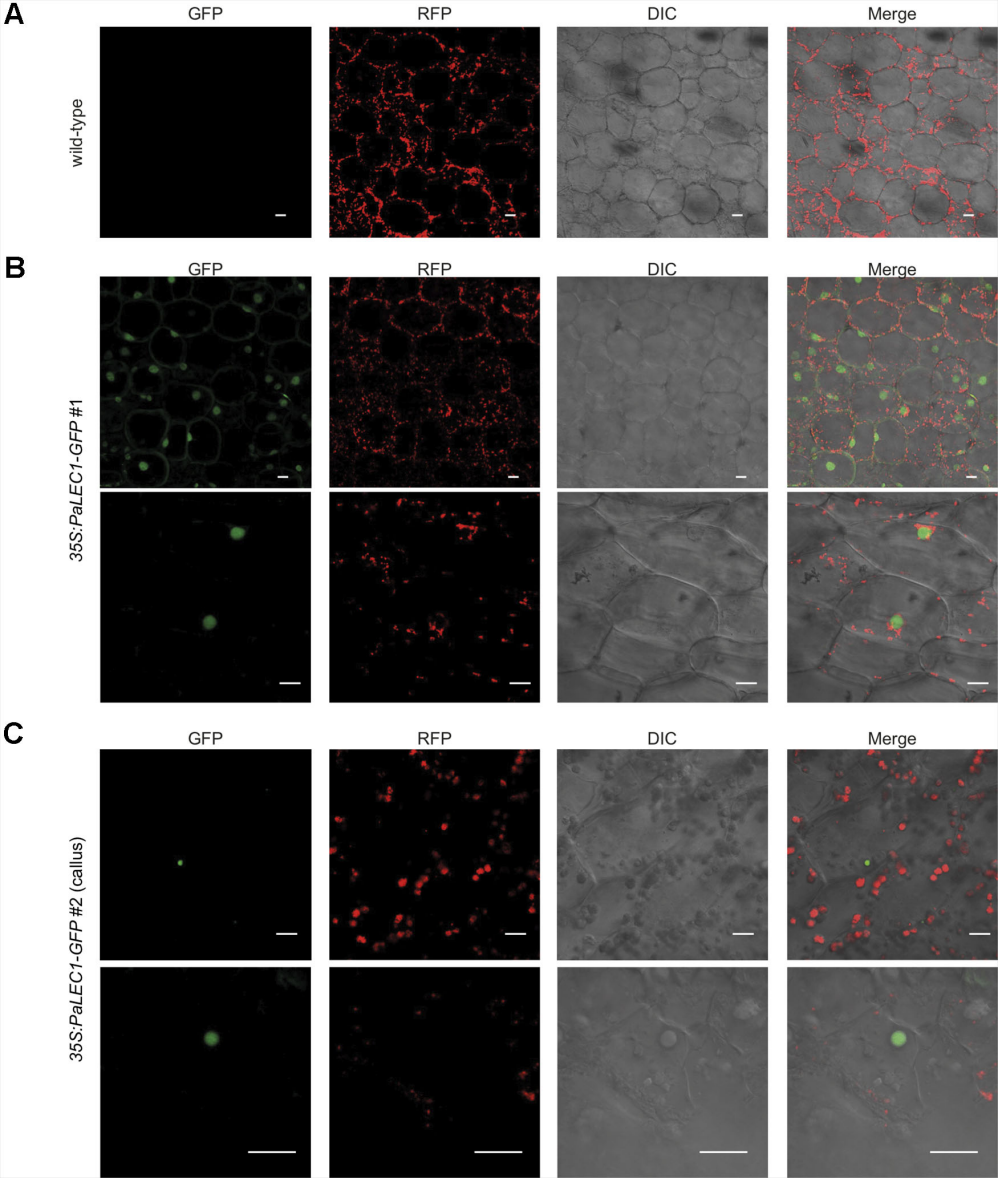
Nuclear localization of PaLEC1-GFP. **(A)** No GFP signal was detected in wild-type plants. **(B)** PaLEC1-GFP was detected in nuclei of cells in roots of *35:PaLEC1-GFP#1* transgenic line. Nuclear localization of PaLEC1-GFP was confirmed in multiple (more than three) independent seedlings. **(C)** PaLEC1-GFP was detected in very few nuclei within callus cells of *35:PaLEC1-GFP#2* transgenic line. This sparsely distribution of nuclear PaLEC1-GFP signal was confirmed in multiple (more than three) protocorm-derived callus tissues. Photos with lower magnification are shown in the upper panel in panels **(B** and **C)**. Photos with higher magnification are shown in the bottom panel in panels **(B** and **C)**. GFP, photographs taken in the GFP channel. RFP, photographs taken in the RFP channel. DIC, differential interference contrast images. Merge, differential interference contrast images of cells superimposed with PaLEC1 (GFP) and chloroplast (RFP) channels. Scale bar = 20 μm.

Nuclear Transcription Factor-Y (NF-Y) is an evolutionarily conserved, DNA-binding, trimeric protein complex composed of NF-YA, NF-YB, and NF-YC subunits (Dolfini et al., 2012). *LEC1* encodes an atypical subunit of the (NF-YB) CCAAT-binding TF. To validate subcellular localization of PaLEC1, confocal microscopy was used to examine the localization of PaLEC1-GFP protein in transgenic plants. As predicted, PaLEC1-GFP was predominantly localized in the nuclei in the cells of the *35S:PaLEC1-GFP#1* line (**Figure 7**). The nuclear localized GFP signal was detected in all of the cells investigated in the *35S:PaLEC1-GFP#1* line. PaLEC1-GFP protein, however, was only detected in a few cells in callus tissues and was therefore much less abundant in *35S:PaLEC1-GFP#2* transgenic line (**Figure 7**), supporting the low expression level shown by western blot. Hence, PaLEC1 protein is a nuclear protein.

It has been reported that *LEC1* regulates many embryonic-specific TFs and storage proteins by directly binding to their promoters through the CCAAT DNA element (Lee et al., 2003; Gnesutta et al., 2017; Pelletier et al., 2017). To investigate whether PaLEC1 activates its targets in the same manner, we retrieved the 1 kb DNA sequences upstream of the start codon of *PeFUS3*, *PeABI3L1*, *PeABI3L2*, *PeBBM*, *PeWRI1*, *Pe7S-1*, *Pe7S-*2, *PeOLE1*, and *PeOEL2* (Cai et al., 2015) and surveyed the presence of the CCAAT element. Because of a large gap within the promoter regions of *PeABI3L2*, it was omitted from further study. Intriguingly, two to five CCAAT DNA elements were found within the 1 kb promoter sequences of *PeFUS3*, *PeABI3L1*, *Pe7S-1*, *Pe7S-*2, and *PeOLE2* (**Supplementary Figure S7**) whose expression was induced by overexpression of PaLEC1 (**Figure 6**). None or only one CCAAT element was present in promoters of *PeBBM*, *PeWRI1*, and *PeOLE1* (**Supplementary Figure S7**) whose expression was not induced in the PaLEC1 overexpressor (**Figure 6**). The correlation between the number of CCAAT elements and activation capability of PaLEC1-GFP suggests that PaLEC1-GFP protein may activate its targets in a CCAAT DNA element dosage-dependent manner.

**Figure 8:**
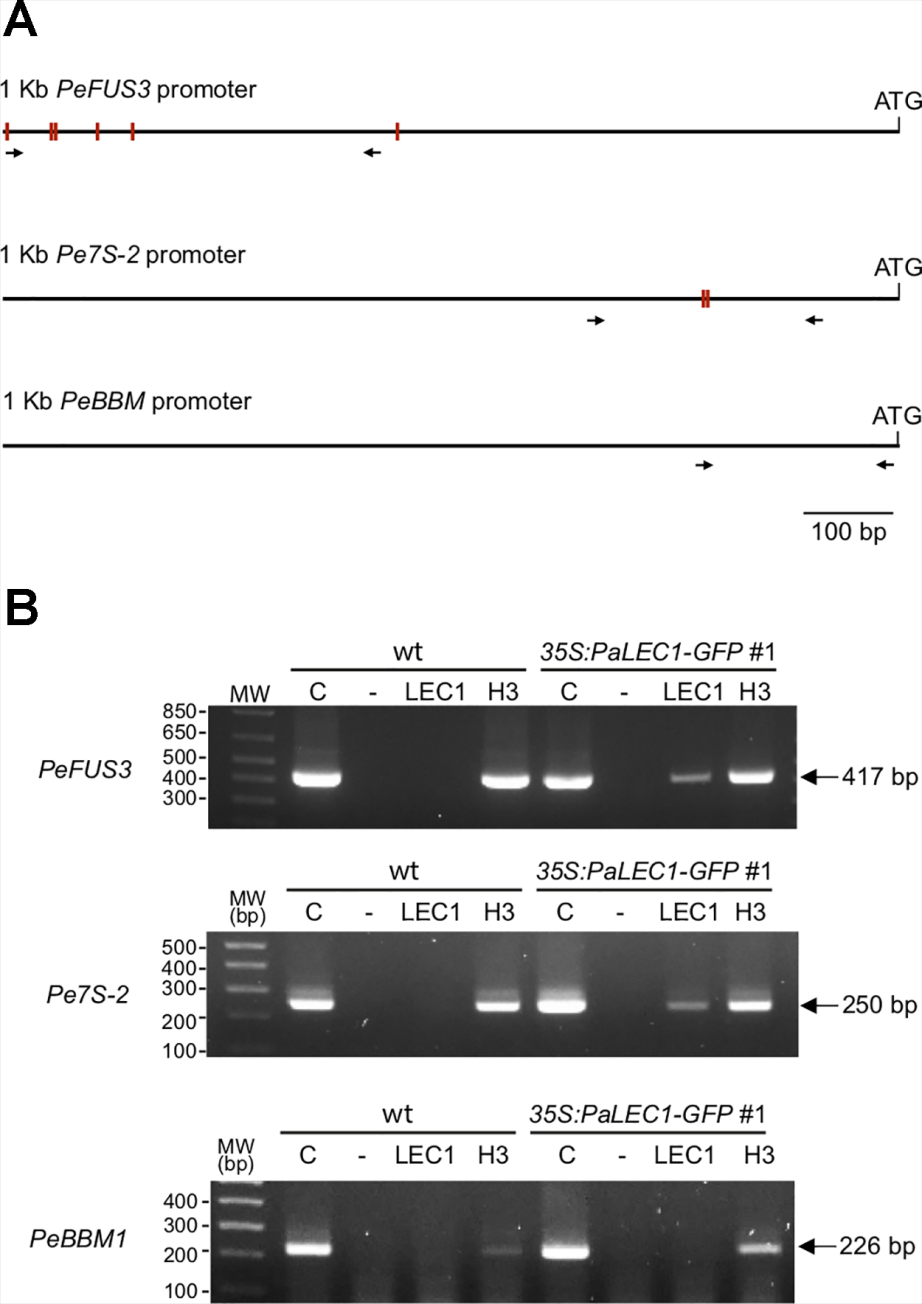
Interaction between PaLEC1-GFP protein and the CCAAT element containing promoters. **(A)** Schematic representation of distribution of the CCAAT elements (red rectangles) in 1 kb upstream of the start codon of *PeFUS3* and *Pe7S-2* genomic DNAs. No CCAAT element was found in 1 Kb promoter region of *PeBBM*. Primers used for PCR reactions were shown as black arrows. **(B)** PCR of chromatin immunoprecipitation isolated DNA fragments to confirm interaction between PaLEC1-GFP protein and the CCAAT-containing promoters of the indicated genes. C, total cell lysate; “-”, no antibody control; PaLEC1, PaLEC1 antibody precipitated chromatin; H3, histone H3 antibody precipitated chromatin. The amplified PCR fragments were verified by DNA sequencing. The same result was validated in two other independent biological samples (**Supplementary Figure S8**).

To test whether PaLEC1-GFP binds the CCAAT element directly, ChIP using PaLEC1 antibody followed by PCR analysis was conducted. Because *PeFUS3* and *Pe7S-2* mRNAs were significantly upregulated by PaLEC1 and tandem CCAAT elements are present in their promoters (**Figure 8** and **Supplementary Figure S8**), *PeFUS3* and *Pe7S-2* were chosen for further ChIP analysis. Histone H3 antibody was used as a positive control and no antibody was included as the negative control. We found that PaLEC1-GFP protein interacted with the *PeFUS3* promoter region containing the CCAAT element but did not interact with *PeBBM* promoter that lacks the CCAAT element (**Figure 8**). Similarly, PaLEC1-GFP was found to interact with the *Pe7S-2* promoter carrying the CCAAT element but not to the *PeBBM* promoter lacking the element (**Figure 8**). These results were confirmed in three independent biological samples. Taken together, it is very likely that PaLEC1 activates *PeFUS3* and *Pe7S-*2 through binding to the CCAAT element-containing promoters.

## Discussion

### PaLEC1 Induces Diverse Somatic Embryonic Structures

Our recent comparative transcriptome study provided evidence that PLB regeneration does not follow the somatic embryogenesis program (Fang et al., 2016) and raises a question about the morphology and anatomy of orchid somatic embryos. In this study, overexpression of PaLEC1 was utilized to induce the somatic embryonic program in *P. equestris*. Similar to PLB induction, active cell division is a prerequisite for *PaLEC1*-induced somatic embryonic structures (**Figures 4B**, **C**). For PLB development, the polarized growth of proliferating cells gives rise to small protuberances with a distinct gradient of cell size, with the smaller cells occupying the future shoot pole and larger and vacuolated cells forming the base of the structure (Lee et al., 2013). For *PaLEC1-*induced somatic embryonic tissues, on the other hand, diverse types of somatic embryonic structures were formed from protocorms or explants of the two *35S:PaLEC1-GFP* transgenic lines regardless of the large difference in the amounts of PaLEC1-GFP protein accumulated. The most common type (~50% in both transgenic lines) was tissue clusters containing multiple shoot apical meristems (multiple-meristem type). For this type of regeneration, multiple meristems were initiated directly from developing protocorms and establishment of unorganized callus tissue did not seem to be a prerequisite. It is important to note that this type of regeneration is very different from direct shoot regeneration during which leaf primordia are generated directly (Košir et al., 2004; Wu and Chen, 2008; Yam and Arditti, 2017). In addition to producing multiple meristems for shoot regeneration, new shoots could also be produced indirectly from callus-type tissue (**Supplementary Figure S2**). The frequency of obtaining the callus-type tissue was higher in the strong *PaLEC1-GFP* overexpressors than in the weak ones (**Table 1**), indicating formation of the embryonic callus may be PaLEC1 dosage-dependent. Dosage-dependent induction of somatic embryogenesis has also been reported for *Arabidopsis* BBM and PLT2 (Horstman et al., 2017b). Intriguingly, the fin-like structure was only observed in the strong overexpressor (*PaLEC1-GFP #1*) and normal protocorm-derived seedlings were only observed in the weak overexpressor (*PaLEC1-GFP #2*). Taken together these results indicate that *PaLEC1*-induced embryonic tissues were morphologically distinct from PLBs. Regardless of the type of structures induced by PaLEC1, all embryonic structures documented here are totipotent and capable of generating clonal orchid seedlings.

### PaLEC1 Activates Part of the Embryonic Program

The embryonic identity of PaLEC1-induced embryonic tissues was verified by expression of embryonic TFs, *PeFUS3*, *PeABI3L1*, *PeABI3L2*, as well as seed storage proteins *Pe7S-1*, and *Pe7S-2*. In *Arabidopsis*, *FUS3*, *ABI3*, and *WRI1* are reported to be the direct targets of LEC1 (Pelletier et al., 2017). However, overexpression of PaLEC1 failed to induce *PeWRI1*. *LEC1* is reported to directly activate *WRI1* or indirectly acts through upregulation of *FUS3* and *LEC2* (Baud et al., 2007; Mu et al., 2008; Shen et al., 2010; Wang and Perry, 2013; Pelletier et al., 2017). Despite a high level of PaLEC1 protein and elevation of *PeFUS3* mRNA in *35S:PaLEC1-GFP*#1 transgenic line, we did not observe induction of *PeWRI1*, suggesting a PaLEC1-independent pathway is required to induce *PeWRI1* in *Phalaenopsis* orchids. Similar to *Arabidopsis* on the other hand, PaLEC1 cannot activate *PeBBM* and only induces a subset of storage proteins (West et al., 1994; Pelletier et al., 2017). In *Arabidopsis*, *BBM* acts upstream of the LEC1-FUS3-ABI3-LEC2 network to induce the somatic embryogenesis program (Horstman et al., 2017b). Whether a similar regulatory module exists in *Phalaenopsis* orchids remains to be tested. Taken together, despite being a potent inducer of somatic embryogenesis in *Phalaenopsis* orchids, the PaLEC1-dependent regulatory network is similar but not identical to that reported in *Arabidopsis*.

*LEC1* belongs to a distinct subgroup of the NF-YB family that is diverged from non-*LEC1*-type NF-YB genes in non-seed vascular plants (Xie et al., 2008). It has been proposed that LEC1-type NF-YB is recruited into a seed-specific regulatory network during evolution. Similar to *Arabidopsis* LEC1-type NF-YB TFs (Calvenzani et al., 2012; Laloum et al., 2013), PaLEC1 also binds to the CCAAT-containing promoters of its downstream genes, *PeFUS3* and *Pe7S-2*. Therefore, PaLEC1 is very likely to regulate its targets by directly acting through binding of the CCAAT DNA element. Even though one CCAAT element was present in the 1 kb promoters of *PeWRI1* and *PeOLE1* genes (**Supplementary Figure S4**), *PeWRI1* and *PeOLE1* mRNAs were not activated by overexpression of PaLEC1-GFP protein, indicating the CCAAT DNA element may not be sufficient for PaLEC1 binding. This notion is supported by studies of *Arabidopsis* and soybean that the G-box (CACGTG), abscisic acid response element (ABRE)-like (C/G/T)ACGTG(G/T)(A/C), and RY (CATGCA) motifs are significantly overrepresented in LEC1 target genes (Pelletier et al., 2017). The involvement of G-box, ABRE-like, and RY motifs in PaLEC1 functions remain to be determined.

In conclusion, our study of PaLEC1-induced somatic embryogenesis has demonstrated that somatic tissues of *Phalaenopsis* orchids do not resemble PLB, a result that is consistent with our previous comparative transcriptome studies. In addition to confirming the role of PaLEC1 in activating the somatic embryonic program, we obtained evidence supporting a direct role for PaLEC1 in controlling its targets and revealed the potential of PaLEC1-induce somatic embryonic tissues for orchid propagation. Conservation of the developmental processes and gene regulatory networks controlled by PaLEC1 is consistent with the notion that LEC1 is a major regulator of embryogenesis.

## Data Availability Statement

All datasets generated for this study are included in the article/**Supplementary Material**.

## Author Contributions

S-CF designed the experiments, coordinated the studies, and wrote the manuscript. J-CC maintained and characterized transgenic orchid plants, and developed the ChIP protocol. J-CC and H-YL carried out the molecular biology experiments. C-GT developed and coordinated the orchid transformation process. All the authors have read and approved the final manuscript.

## Funding

This work was supported by the Innovative Translational Agricultural Research Grants (to S-CF); and in part by a grant (to S-CF) from the Biotechnology Center in Southern Taiwan, Academia Sinica.

## Conflict of Interest

The authors declare that the research was conducted in the absence of any commercial or financial relationships that could be construed as a potential conflict of interest.
