# Supplementary figures and images for: Phalaenopsis LEAFY COTYLEDON1-Induced Somatic Embryonic Structures Are Morphologically Distinct From Protocorm-Like Bodies
Source: Front Plant Sci. 2019 Nov 29;10:1594. doi: 10.3389/fpls.2019.01594 (PMC6896055; doi:10.3389/fpls.2019.01594)

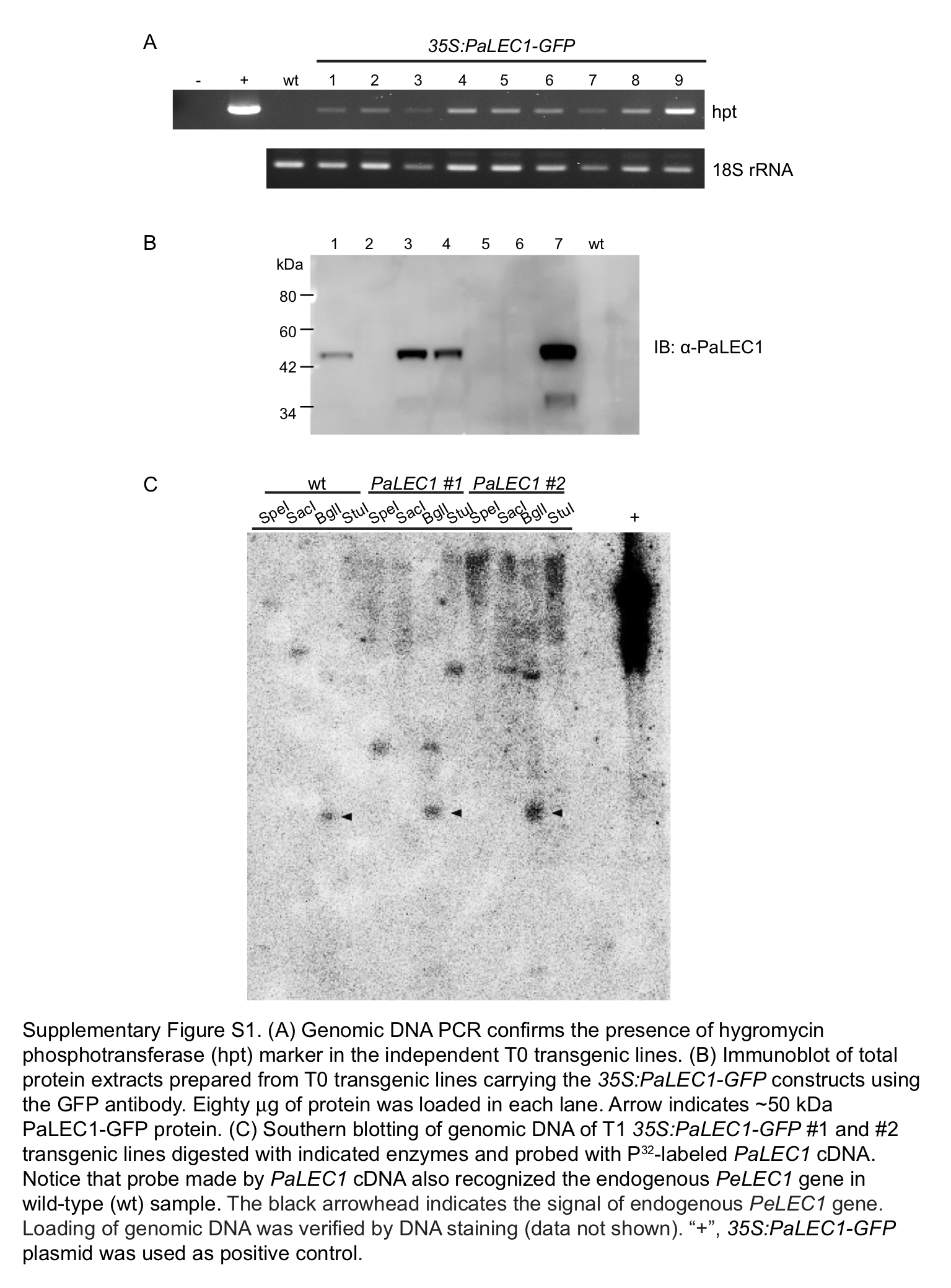

Supplement: Supplementary file 1 [file Image_1.tiff]

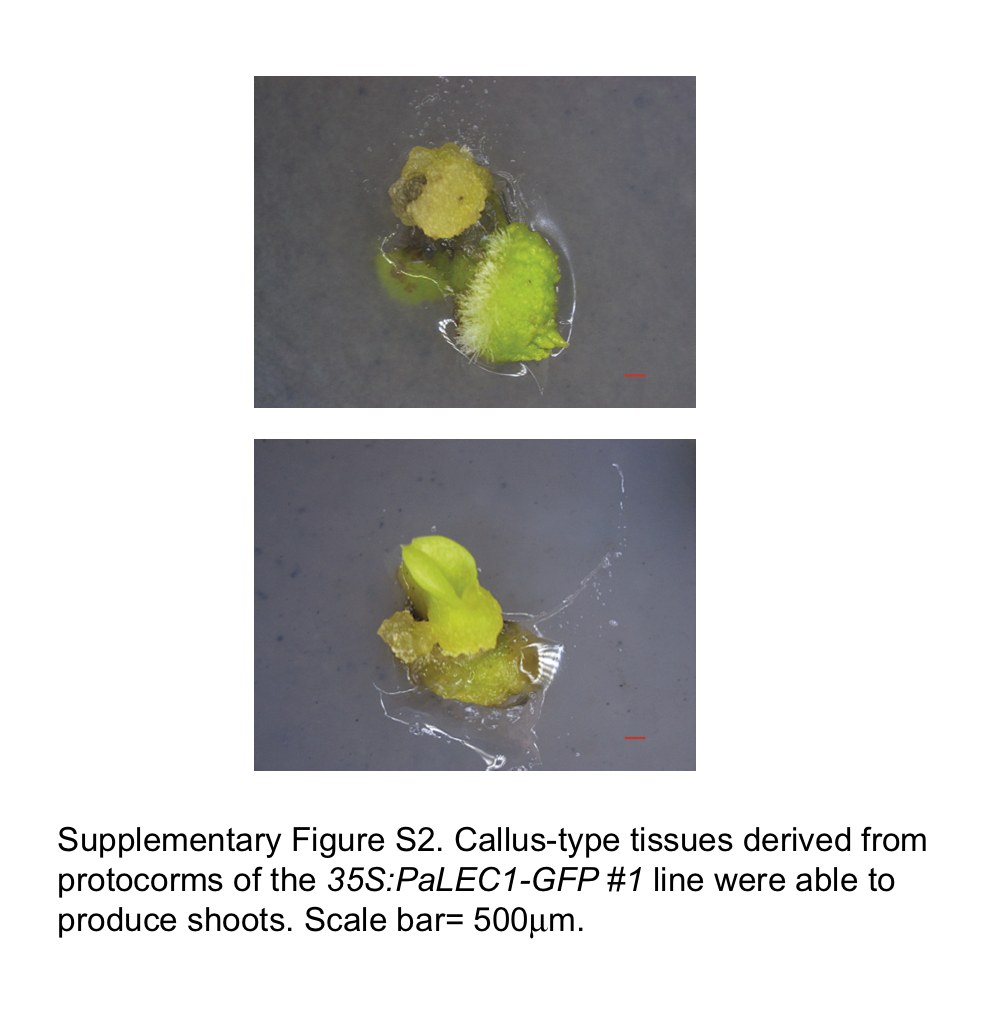

Supplement: Supplementary file 2 [file Image_2.tiff]

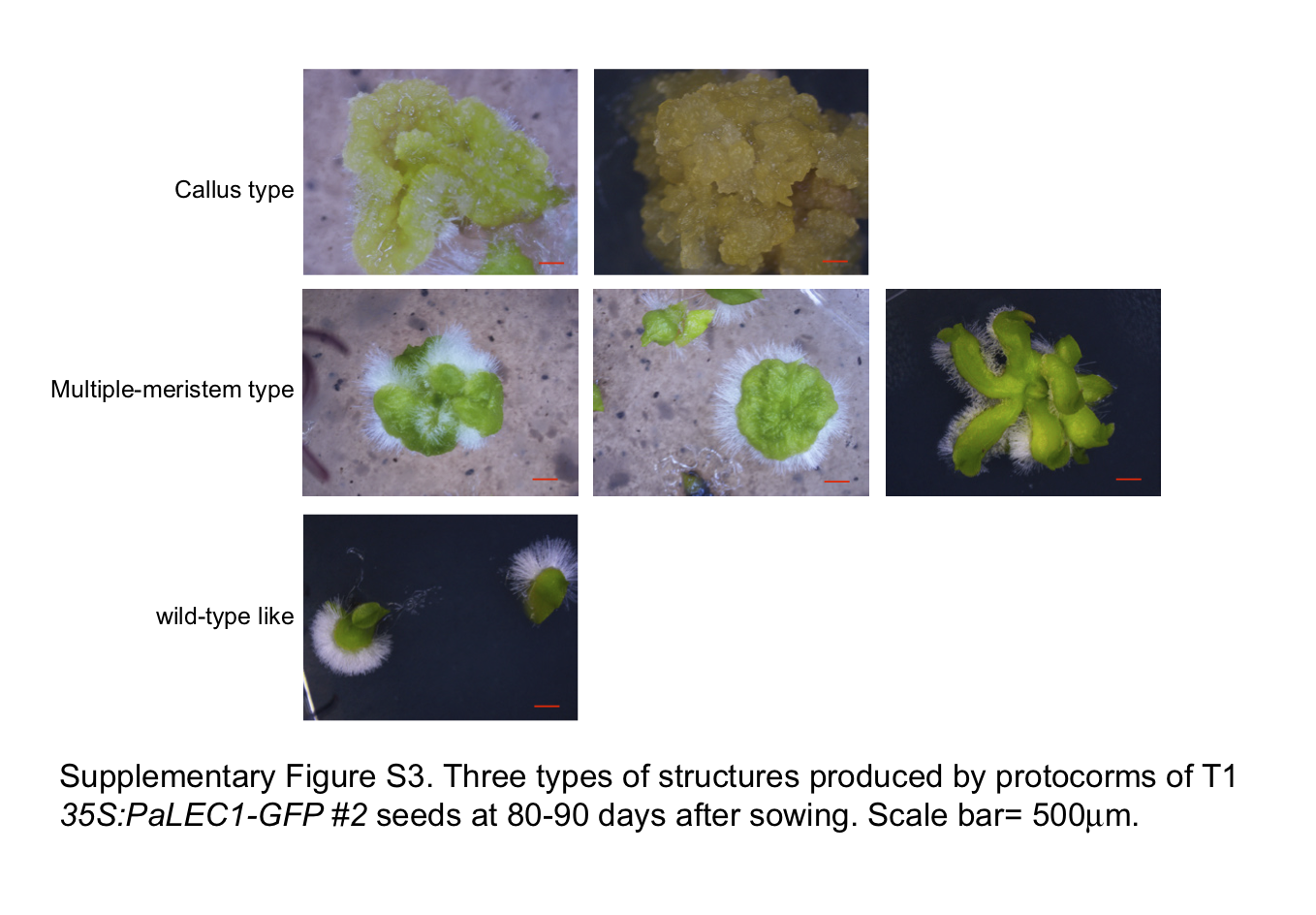

Supplement: Supplementary file 3 [file Image_3.tiff]

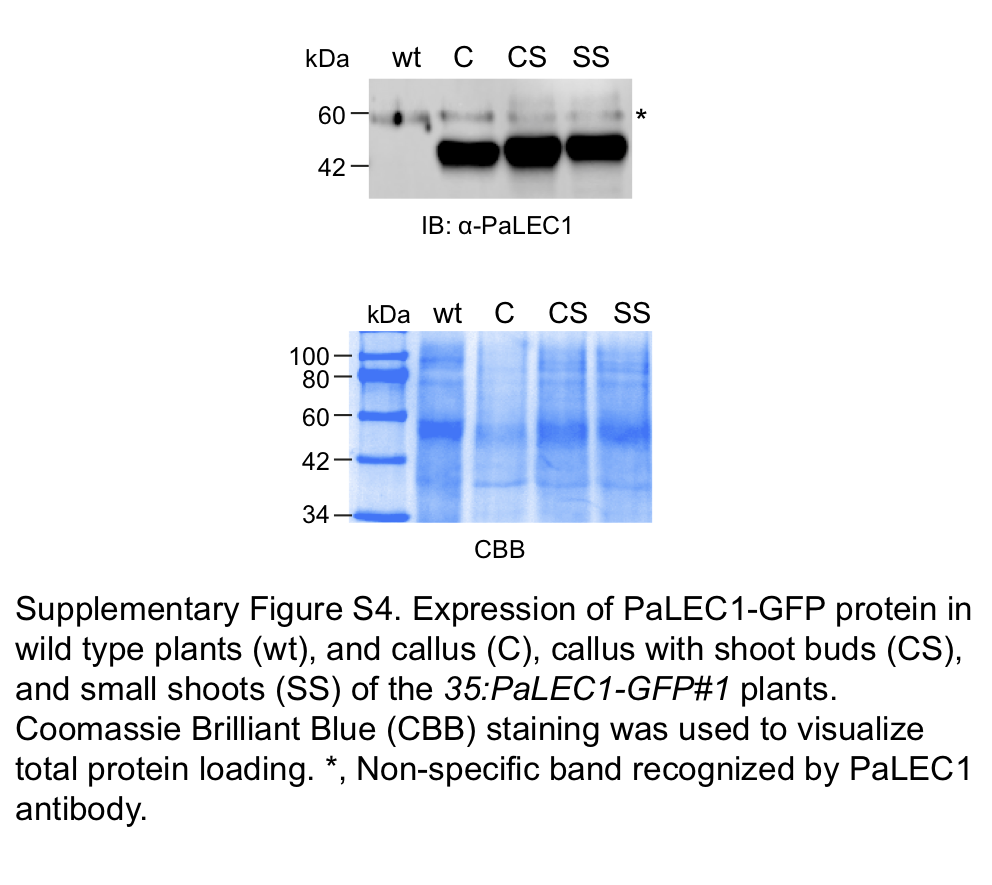

Supplement: Supplementary file 4 [file Image_4.tiff]

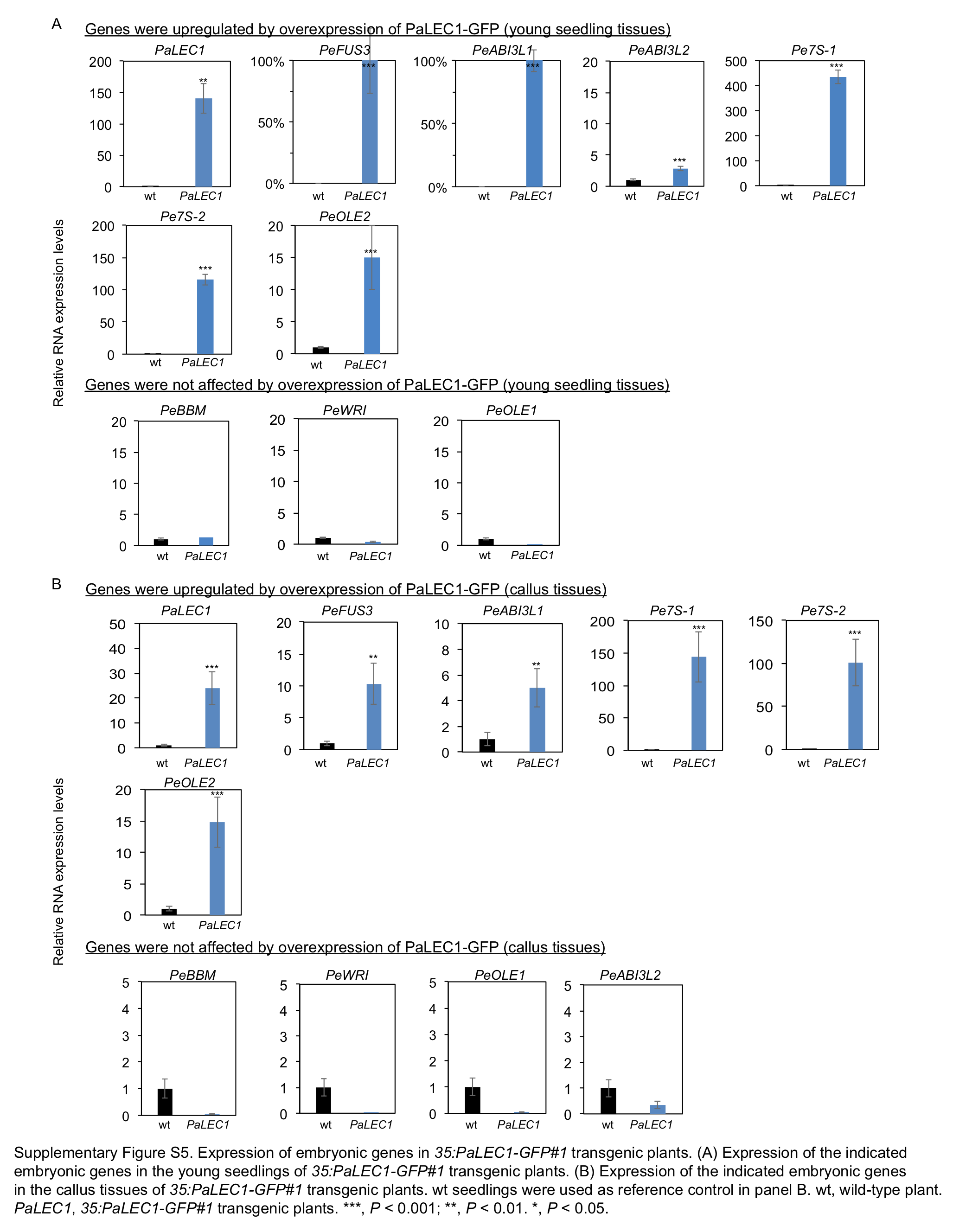

Supplement: Supplementary file 5 [file Image_5.tiff]

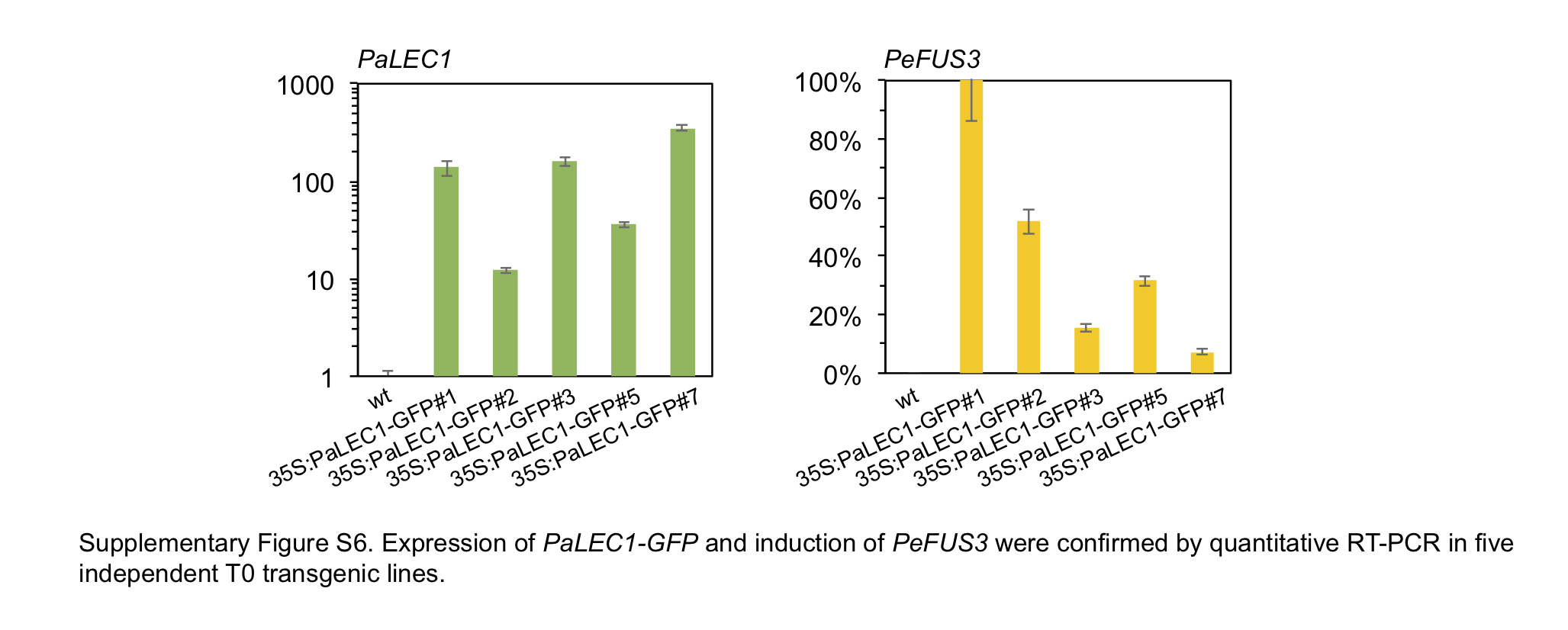

Supplement: Supplementary file 6 [file Image_6.tiff]

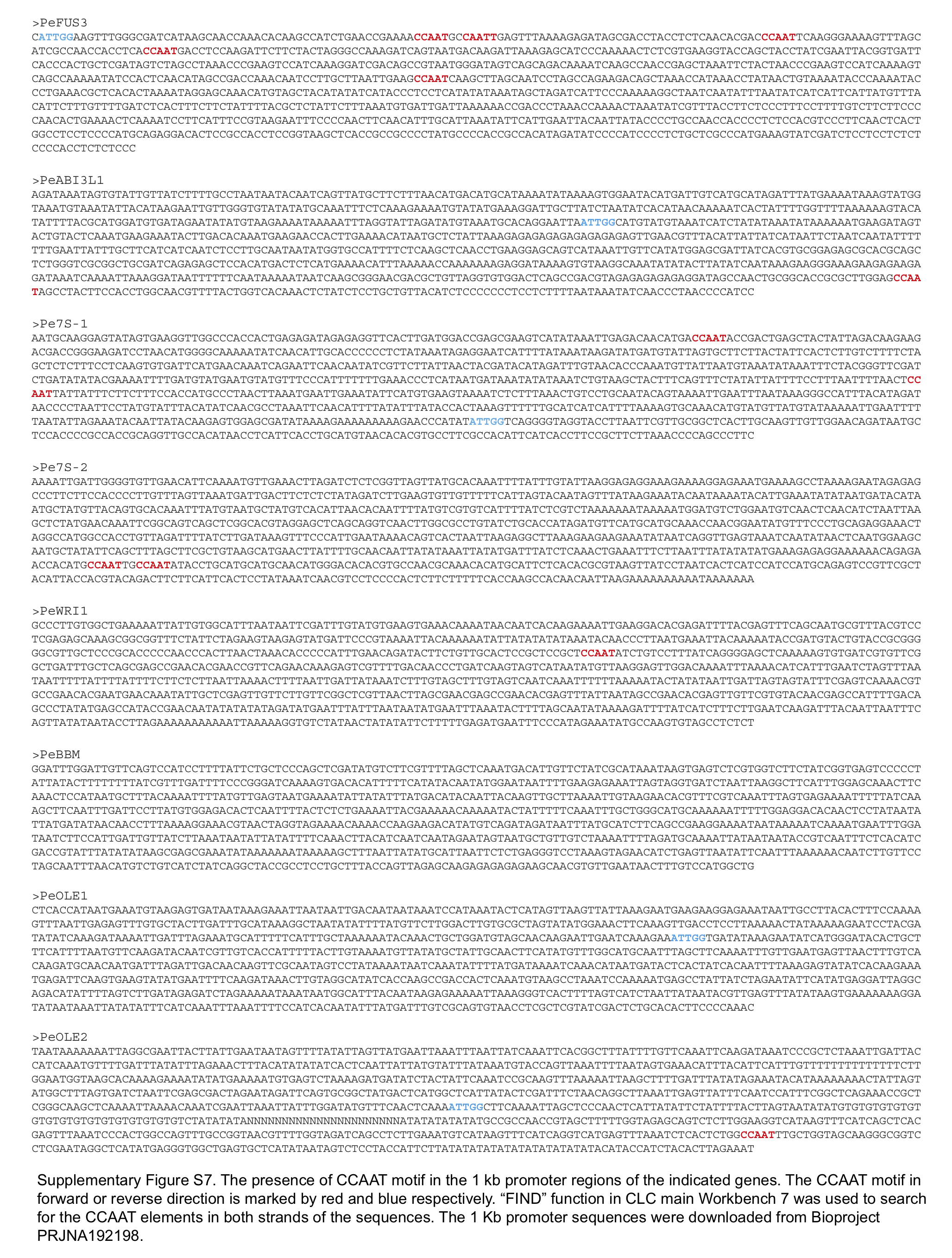

Supplement: Supplementary file 7 [file Image_7.tiff]

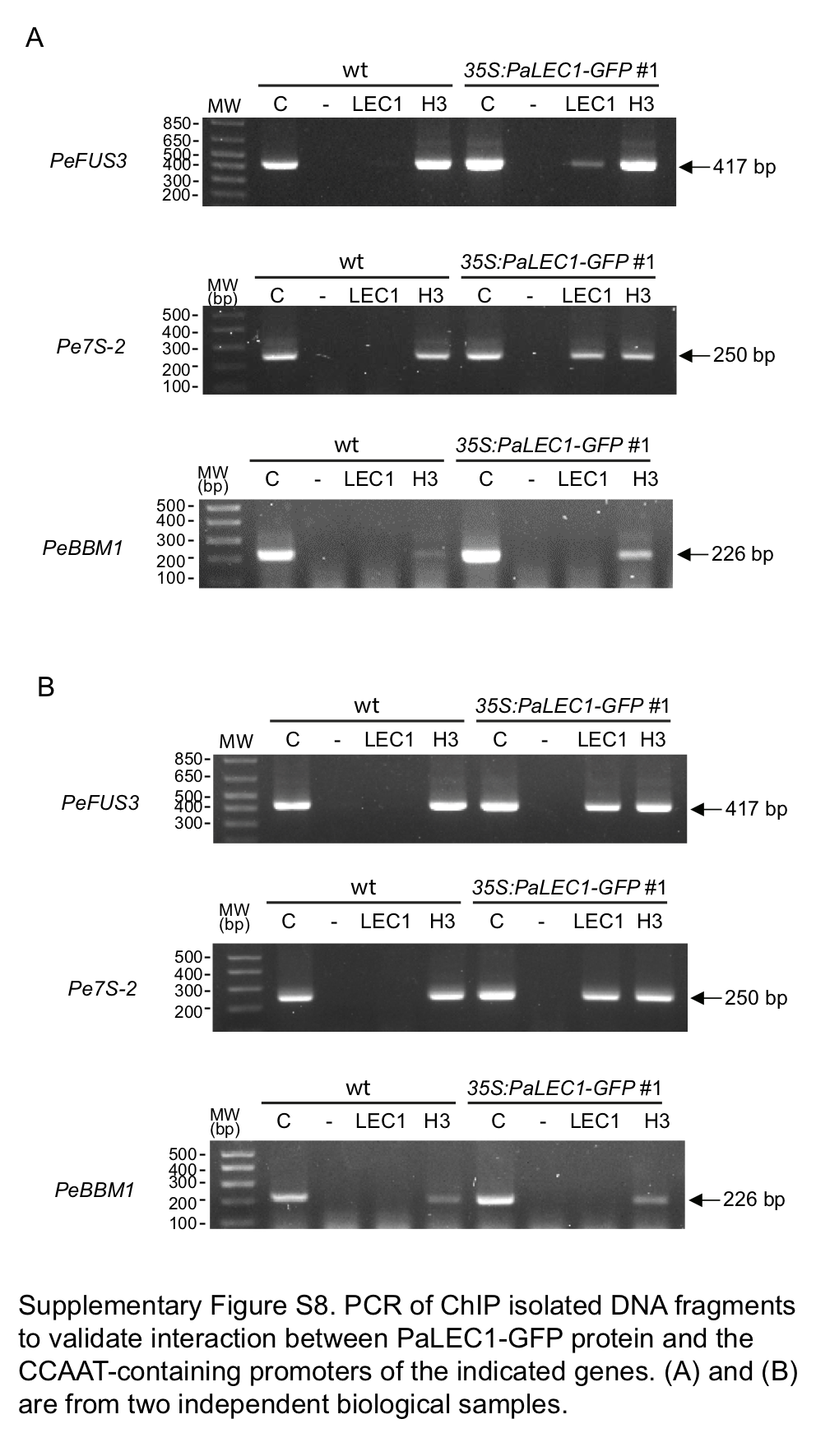

Supplement: Supplementary file 8 [file Image_8.tiff]
