# Supplementary material for: Phalaenopsis LEAFY COTYLEDON1-Induced Somatic Embryonic Structures Are Morphologically Distinct From Protocorm-Like Bodies
Source: Front Plant Sci. 2019 Nov 29;10:1594. doi: 10.3389/fpls.2019.01594 (PMC6896055; doi:10.3389/fpls.2019.01594)
Supplement: Supplementary file 9 [file Table_1.docx]

Supplementary Table S1. List of primer pairs used for qRT-PCR and ChIP-PCR.

| Gene name | Forward primer | Reverse primer | Amplicon size (bp) | Annealing temp (°C) |
| --- | --- | --- | --- | --- |
| RT-PCR | | | | |
| *PeABI3L1* | 5′-GGGGATTTTGTGAAAGCAAA-3′ | 5′-TATTGCTCGCTCCCTTCATT-3′ | 136 | 58 |
| *PeABI3L2* | 5′-TGTTAACTCAGGAAAGGCTCATC-3′ | 5′-AATATTCTCCCAAAGCACAGAGAG-3′ | 113 | 62 |
| *PeBBM* | 5′-CTTTTGGGCAAAGGACTTCA-3′ | 5′-ATAAGCCCTTGCTGCCTTTT-3′ | 186 | 58 |
| *PeFUS3* | 5′-AGGCCAATCTTCCAATCCTT-3′ | 5′-GGCTTCTCACAAATTCACCAG-3′ | 156 | 62 |
| *PeLEC1* | 5′-AGCTCGGCTTTGACGACTAC-3′ | 5′-CGACGGAAGATGATACGACA-3′ | 143 | 62 |
| *PeOLE1* | 5′-GAGTAGGAGATCGCGGACAA-3′ | 5′-ACAGGGCTGAAAATGACGAG-3′ | 178 | 60 |
| *PeOLE2* | 5′-ACGTCGTTATCTTGGATCGTG-3′ | 5′-CTATGGCCCACATCTCTGGT-3′’ | 146 | 58 |
| *Pe7S-1* | 5′-TATTGCTCGCTCCCTTCATT-3′ | 5′-CTCTCTCCCTGCCACGTAAG-3′ | 145 | 58 |
| *Pe7S-2* | 5′-AACAGAGGCAAGCTCCTTGA-3′ | 5′-CCTGAAAGCTCGTGTGTTGA-3′ | 132 | 58 |
| *PeWRI1* | 5′-TTGTGTTGTGGAGGAACTCG-3′ | 5′-CAAATTCGGAAGGTCTCCAA-3′ | 182 | 58 |
| *PeUBI* | 5′-AACTCCATCGCCTTCCTCTT-3′ | 5′-TGAAGCATGGCATCAATTTC-3′ | 101 | 58 to 62 |
| ChIP-PCR | | | | |
| *PeFUS3_pro* | 5′-GGAAGTTTGGGCGATCATAA-3′ | 5′-AATTGGGTCGTGTTGAGAGG-3′ | 417 | 60 |
| *Pe7S-2_pro* | 5′-AGCTTCGCTGTAAGCATGAA-3′ | 5′-ACGTGGTAATGTAGCGAACG-3′ | 250 | 58 or 60 |
| *PeBBM_pro* | 5′-ACCGTCAATTTCTCACATCG-3′ | 5′-CGTTGCTTCTCTCTCTCTTGC-3′ | 226 | 58 or 60 |
